# Machine Learning‐Based Glycolipid Metabolism Gene Signature Predicts Prognosis and Immune Landscape in Oesophageal Squamous Cell Carcinoma

**DOI:** 10.1111/jcmm.70434

**Published:** 2025-03-22

**Authors:** Lin Zhu, Feng Liang, Xue Han, Bin Ye, Lei Xue

**Affiliations:** ^1^ Department of Oncology The Affiliated Suqian First People's Hospital of Nanjing Medical University Suqian China; ^2^ Department of Gastroenterology, Huai'an Second People's Hospital The Affiliated Huai'an Hospital of Xuzhou Medical University Huai'an China; ^3^ Department of Thoracic Surgery The First Affiliated Hospital of Nanjing Medical University Nanjing China

**Keywords:** glycolipid metabolism, machine learning, MECP2, oesophageal squamous cell carcinoma, tumour immune microenvironment

## Abstract

Using machine learning approaches, we developed and validated a novel prognostic model for oesophageal squamous cell carcinoma (ESCC) based on glycolipid metabolism‐related genes. Through integrated analysis of TCGA and GEO datasets, we established a robust 15‐gene signature that effectively stratified patients into distinct risk groups. This signature demonstrated superior prognostic value and revealed significant associations with immune infiltration patterns. High‐risk patients exhibited reduced immune cell infiltration, particularly in B cells and NK cells, alongside increased tumour purity. Single‐cell RNA sequencing analysis uncovered unique cellular composition patterns and enhanced interaction intensities in the high‐risk group, especially within epithelial and smooth muscle cells. Functional validation confirmed MECP2 as a promising therapeutic target, with its knockdown significantly inhibiting tumour progression both in vitro and in vivo. Drug sensitivity analysis identified specific therapeutic agents showing potential efficacy for high‐risk patients. Our study provides both a practical prognostic tool and novel insights into the relationship between glycolipid metabolism and tumour immunity in ESCC, offering potential strategies for personalised treatment.

## 1 Background

In 2020, approximately 604,000 new cases of oesophageal cancer and 544,000 related deaths were reported worldwide. The corresponding age‐standardised incidence and mortality rates were 6.3 and 5.6 per 100,000, respectively. If current trends persist, the global burden is projected to rise to 957,000 new cases and 880,000 deaths by 2040, underscoring the critical threat this disease poses to public health. Moreover, oesophageal squamous cell carcinoma (ESCC) represents approximately 85% of all oesophageal cancer cases [1].

A hallmark of cancer development is metabolic reprogramming, wherein cancer cells exhibit distinct metabolic profiles compared to normal cells. These alterations, which include enhanced glucose metabolism and dysregulated amino acid and lipid pathways, enable rapid tumour proliferation and survival under hostile microenvironmental conditions [2, 3]. The Warburg effect has been extensively reported, and its content has become an indisputable fact [4]. Although this process generates less energy per molecule of glucose than oxidative phosphorylation, it allows for faster ATP production, particularly under conditions of high glucose availability [5]. Additionally, the lactate produced through glycolysis can suppress immune responses and promote invasion, metastasis and tissue destruction [6].

In parallel, aberrant lipid metabolism has emerged as a key component of cancer biology [7, 8]. Lipid metabolites such as fatty acids and sphingosines not only provide energy but also regulate the immune microenvironment and tumour angiogenesis. For instance, fatty acid metabolites can activate signalling pathways such as PI3K/Akt and MAPK, enhancing chemoresistance and tumour growth [9, 10]. Moreover, prostaglandin E2 (PGE2), a product of lipid metabolism, has been shown to suppress antitumour immunity by inhibiting T cells and natural killer (NK) cells while promoting the polarisation of tumour‐associated macrophages into immunosuppressive M2 phenotypes [11, 12]. Fatty acids and other lipid metabolites can promote tumour angiogenesis by activating angiogenesis‐related signalling pathways (e.g., VEGF pathway), providing more oxygen and nutrients to the tumour [13, 14]. In addition, fatty acid synthesis is often enhanced in tumour cells to meet their needs for membrane structure and signalling molecules. The fatty acid synthesis pathway is usually controlled by key enzymes such as fatty acid synthase (FASN). FASN is overexpressed in many types of cancer (such as ovarian cancer, liver cancer and colon cancer), and its increased activity enables tumour cells to obtain more fatty acids for the synthesis of membrane phospholipids, cholesterol and other lipids [15].

In recent years, machine learning technologies have demonstrated enormous potential in cancer research, particularly in prognostic prediction and biomarker identification [16, 17, 18, 19]. Advanced algorithms such as deep learning and ensemble learning can extract meaningful patterns from complex gene expression data and establish accurate prediction models [20, 21, 22, 23]. In cancer prognosis research, machine learning methods have been successfully applied to multiple cancer types, including breast cancer, lung cancer and colorectal cancer, significantly improving prediction accuracy and clinical utility. These methods not only process high‐dimensional genomic data but also integrate multi‐omics information, providing crucial support for personalised treatment decisions.

Despite growing evidence linking metabolic pathways to cancer progression, prognostic models focusing on glycolipid metabolism genes in ESCC remain underdeveloped. In this research, we employed bioinformatics approaches to pinpoint genes linked to glycolipid metabolism that are relevant to the prognosis of ESCC. By constructing a risk assessment model, we not only underscored the prognostic value of these genes but also uncovered potential avenues for therapeutic interventions in the treatment of ESCC.

## 2 Methods

### 2.1 Data Acquisition and Preprocessing

The study utilised comprehensive genomic data obtained from two major repositories: The Gene Expression Omnibus (GEO) and The Cancer Genome Atlas (TCGA) database. Specifically, we integrated the TCGA‐ESCC dataset with the GSE53624 dataset from GEO. To ensure data compatibility and minimise technical variability between different platforms, we employed the R package ‘sva’ implementing an empirical Bayes framework for batch effect correction. Following rigorous quality control and data preprocessing, the harmonised datasets were merged to create a robust analytical framework for subsequent investigations [24, 25].

### 2.2 Development of the Prognostic Model

To pinpoint glycolipid metabolism‐related genes with prognostic relevance, we conducted a univariate Cox regression analysis on the integrated dataset. Genes with a significant association with overall survival were subjected to further refinement using Lasso regression analysis, followed by multivariate Cox regression modelling [26]. This process allowed us to establish a prognostic risk scoring system. The median risk score was used to stratify patients into high‐ and low‐risk groups for subsequent analysis.

### 2.3 Differential Expression and Pathway Enrichment Analysis

To identify differentially expressed genes (DEGs) between high‐ and low‐risk groups, the R package limma was utilised, with selection thresholds set at adjusted *p*‐values < 0.01 and |log(FoldChange)| > 0.5 [27]. Biological processes and molecular functions linked to the DEGs were investigated through Gene Ontology (GO) enrichment analysis using the ‘clusterProfiler’ package. A *p*‐value < 0.05 was considered statistically significant, and the findings were visualised with the ggplot2 package.

### 2.4 Immune Cell Infiltration Analysis

The composition and distribution of immune cell populations in tumour samples from high‐ and low‐risk groups were assessed using several computational algorithms, such as CIBERSORT, EPIC, MCPcounter, Quantiseq and xCell [28, 29, 30, 31]. Spearman's correlation analysis was performed to examine the relationships between the risk score and immune‐related metrics, including tumour purity, ESTIMATE score, immune score and stromal score.

### 2.5 Analysis of Gene Variation

To comprehensively characterise genetic alterations, we conducted detailed analyses of both mutation spectra and frequencies across tumour samples. The mutation spectrum analysis encompassed a systematic evaluation of various mutation types and their distribution patterns across the genome, while mutation frequency assessment quantified the prevalence of specific genetic alterations within our study cohort. Utilising the ‘Maftools’ package, we performed sophisticated computational analyses to examine and visualise these genetic variations. This analytical framework enabled us to investigate the relationships between genetic alterations and key tumour characteristics, including tumour purity, ESTIMATE score, immune score and stromal score [32]. Furthermore, we leveraged the ‘oncoplot’ function within ‘Maftools’ to generate comprehensive mutation landscape visualisations and waterfall plots, providing detailed representations of tumour mutation burden (TMB) across samples.

### 2.6 Single‐Cell RNA Sequencing (scRNA‐Seq) Analysis

scRNA‐seq data for oesophageal cancer were retrieved from the GSE160269 dataset. Using the ‘Seurat’ package [33, 34], scRNA‐seq data were normalised and processed. Data visualisation tools such as PCA and t‐SNE were used to perform dimensionality reduction and clustering. Cell subpopulations were annotated based on marker gene expression. Intercellular communication networks were inferred and analysed using the CellChat package [35, 36].

### 2.7 Drug Sensitivity Profiling

The CellMiner database compiles extensive drug sensitivity data [37]. This platform aims to assist users in analysing the responses of cancer cell lines to different drugs and correlating them with biological information such as gene expression data, mutation data and gene copy number changes, thereby exploring potential drug sensitivity and resistance mechanisms. Correlations between drug sensitivity and the risk score were assessed using Pearson's correlation analysis.

### 2.8 Cell Culture

The TE1 and MEC25 cell lines were sourced from the Cell Bank of the Chinese Academy of Sciences. When cell confluency exceeded 80%, passaging was performed. All cells were maintained at 37°C in a humidified atmosphere with 5% carbon dioxide. The target sequences used for constructing stable lentiviral knockdown cell lines were as follows: MECP2 (human): GAAAGAAGAGAAAGAGGGCAA and MECP2 (mouse): CGCTCTAAAGTAGAATTGATT.

### 2.9 Reverse Transcription‐Quantitative Polymerase Chain Reaction (RT‐qPCR)

Total RNA was extracted using TRIzol reagent. After 24 h of cell seeding, cDNA was synthesised via reverse transcription using a PrimeScript RT Reagent Kit (Takara Bio Inc., RR047A). Quantitative PCR (qPCR) analysis was performed on a QuantStudio 3 Real‐Time PCR System (Thermo Fisher Scientific) using the SYBR‐based TB Green Premix Ex Taq II kit (Takara Bio Inc., RR820A). The primer sequences used were as follows: MECP2 (human): Forward: TGAAGGCTGGACACGGAAGCTT; Reverse: CAGGGATGTGTCGCCTACCTTT. MECP2 (mouse): Forward: CAAAGGAAGTCTGGCCGATCTG; Reverse: CATTAGGGTCCAAGGAGGTGTC.

### 2.10 CCK‐8 Assay

Cells were plated in 96‐well plates at a density of 2000 cells per well and allowed to adhere. After 24, 48 or 72 h, 10 μL of CCK‐8 reagent was added to each well and mixed gently. The plates were incubated in the dark at 37°C for 2 h, and the absorbance at 450 nm was measured using a microplate reader.

### 2.11 Colony Formation Assay

Single‐cell suspensions were seeded into six‐well plates at a density of 800 cells per well. After 10 days of culture, the medium was gently removed, and the cells were fixed with 4% paraformaldehyde (PFA) at room temperature for 20 min, followed by two PBS washes. Cells were then stained with 0.1% crystal violet solution (or other suitable staining reagents) for 20 min at room temperature. Excess stain was gently rinsed off with running water until the background was clear, and the plates were air‐dried. The stained colonies were then counted for analysis.

### 2.12 Animal Experiment

C57BL/6 female mice, 6–8 weeks old, were housed under specific pathogen‐free (SPF) conditions. MEC25 cells during their logarithmic growth phase were collected, rinsed with PBS and resuspended as a single‐cell solution with a density of 5 × 10^6^ cells per millilitre. Following skin disinfection with alcohol, a sterile syringe was utilised to administer a subcutaneous injection of 100 μL of the cell suspension into the dorsum of each mouse. Tumour development was monitored every 2–3 days by measuring the tumour's length (*L*) and width (*W*), and the volume was calculated using the formula: Volume = *L* × *W* ^2^/2. Mice were euthanised by cervical dislocation when the tumour volume reached around 1000 mm^3^.

### 2.13 Statistical Analysis

All statistical analyses were conducted using R (version 4.4.2). Statistical comparisons between groups were conducted utilising either Student's *t* test or the Wilcoxon rank‐sum test. Correlations were assessed using the Spearman method. Results with *p*‐values < 0.05 were considered statistically significant.

## 3 Results

### 3.1 Building and Testing a Prognostic Model for ESCC

**FIGURE 1:**
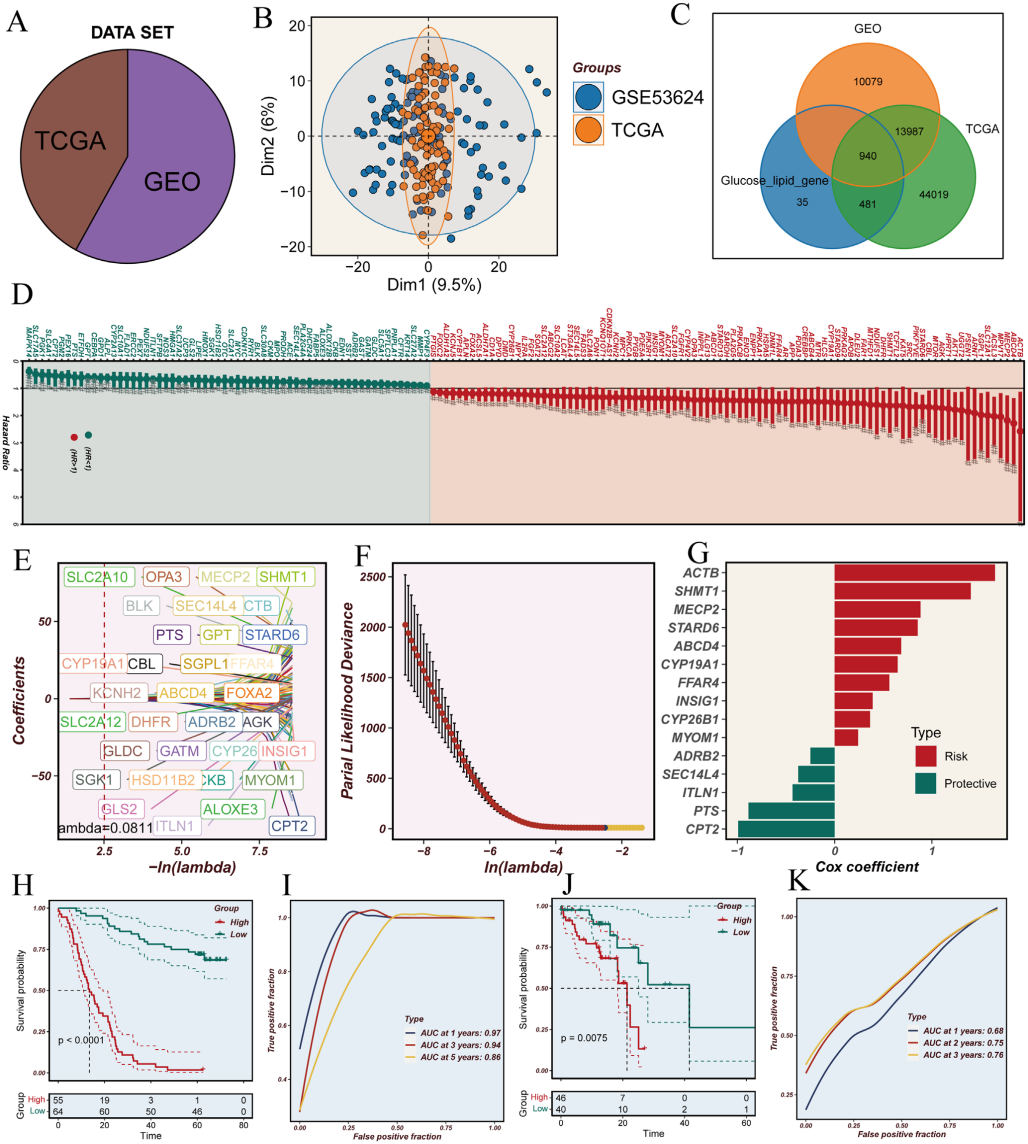
Development and validation of a glycolipid metabolism‐based prognostic model in ESCC using TCGA and GEO datasets. (A) Scale plots demonstrating batch effect correction between TCGA (*n* = 86) and GEO (*n* = 119) datasets. (B) Principal component analysis (PCA) plots illustrating the distribution of samples from integrated TCGA and GEO cohorts. (C) Venn diagram depicting the intersection of genes between TCGA, GEO datasets and known glycolipid metabolism‐related genes. (D) Forest plot of prognostic genes identified through univariate Cox regression analysis. (E, F) LASSO regression analysis for the identification of prognostically significant gene signatures. (G) Multivariate Cox regression analysis revealing independent prognostic factors. (H, I) Kaplan–Meier survival curves and time‐dependent ROC curves demonstrating the predictive performance of the risk stratification model in the GEO cohort. (J, K) Validation of the prognostic model in the TCGA cohort through survival analysis and time‐dependent ROC curves.

Following batch effect elimination, we successfully integrated the GSE53624 (*n* = 119) and TCGA‐ESCC (*n* = 86) datasets (Figure 1A,B). Through systematic intersection analysis with glycolipid metabolism‐related genes, we identified 940 genes common to both datasets (Figure 1C). Initial prognostic analysis revealed a subset of genes significantly associated with patient outcomes (Figure 1D). To develop a robust prognostic model, we employed a rigorous statistical approach combining LASSO and multivariate Cox regression analyses. This methodology identified 15 key glycolipid metabolism genes with significant prognostic values: ACTB, SHMT1, MECP2, STARD6, ABCD4, CYP19A1, FFAR4, INSIG1, CYP26B1, MYOM1, ADRB2, SEC14L4, ITLN1, PTS and CPT2 (Figure 1E–G).

Comprehensive survival analyses revealed striking differences between risk groups, with high‐risk patients demonstrating significantly poorer outcomes across both the GEO and TCGA cohorts (Figure 1H,J). The prognostic model exhibited robust predictive capability, achieving remarkable performance in the GEO dataset, with area under the curve (AUC) values of 0.97, 0.94 and 0.86 for 1‐, 3‐ and 5‐year survival predictions, respectively. Similar validation in the TCGA dataset yielded AUC values of 0.68, 0.75 and 0.76 for the corresponding time points (Figure 1I,K).

### 3.2 Immunophenotypic Analysis Between Different Risk Groups

**FIGURE 2:**
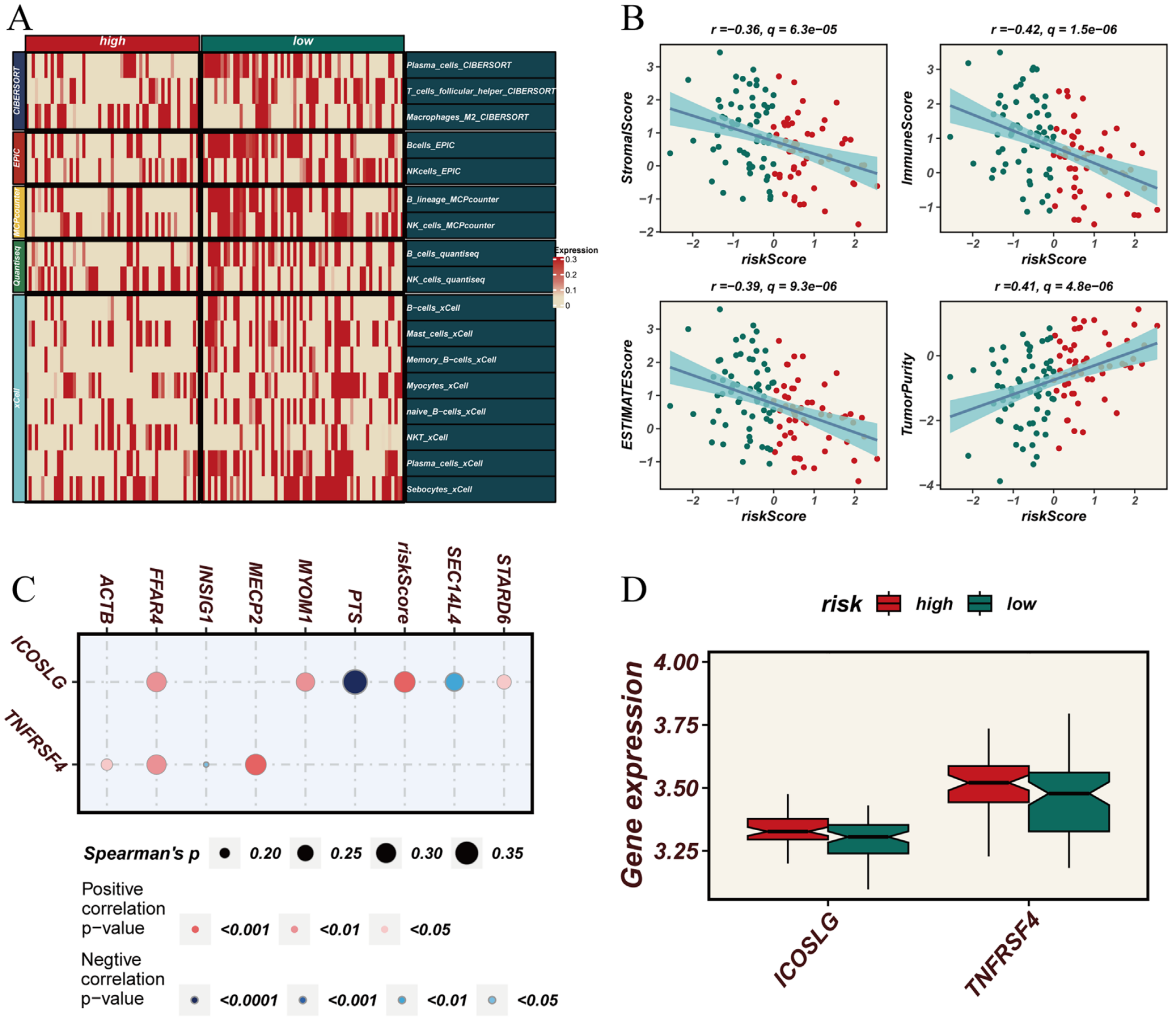
Immunoinfiltration profiles exhibited distinct characteristics across various risk score groups. (A) Multi‐algorithm (CIBERSORT, EPIC, MCPcounter, Quantiseq and xCell) analysis of different immune cell infiltration in the high‐risk or low‐risk score groups. (B) Correlation analysis of StromalScore, ImmuneScore, ESTIMATEScore, Tumorpurity and risk score. (C) Correlation analysis of immunoregulatory molecules ICOSLG and TNFRSF4 with part of selected glycolipid metabolism genes. (D) Expression of immunoregulatory molecules ICOSLG and TNFRSF4 in the high‐risk or low‐risk score groups.

We used multiple algorithms such as CIBERSORT, EPIC, MCPcounter, Quantiseq and xCell to evaluate the types and distribution of immune cells in tumour samples of high‐ and low‐score groups. Figure 2A demonstrates that B cells and NK cells exhibited a declining trend in the high expression group of glycolipid metabolism genes, as indicated by multiple algorithms. Additionally, M2 macrophages, mast cells, memory B cells, naive B cells and natural killer T cells also showed the same trend in CIBERSORT and xCell analysis results. ESTIMATEScore, ImmuneScore and StromalScore were negatively correlated with the risk score, while TumorPurity was positively correlated. This indicates that the higher the risk score, the higher the tumour purity and the lower the immune score (Figure 2B). Further analysis revealed that genes FFAR4, MYOM1 and STARD6 were positively correlated with the co‐stimulatory molecule ICOSLG expressed on immune cells, while PTS and SEC14L4 were negatively correlated. Additionally, ACTB, FFAR4 and MECP2 were positively correlated with the co‐stimulatory molecule TNFRSF4 expressed on T cells (Figure 2C). Overall, co‐stimulatory molecules ICOSLG and TNFRSF4 were overexpressed in the high‐risk corner (Figure 2D).

### 3.3 Exploration of Mutation Profiles and Biological Mechanisms

**FIGURE 3:**
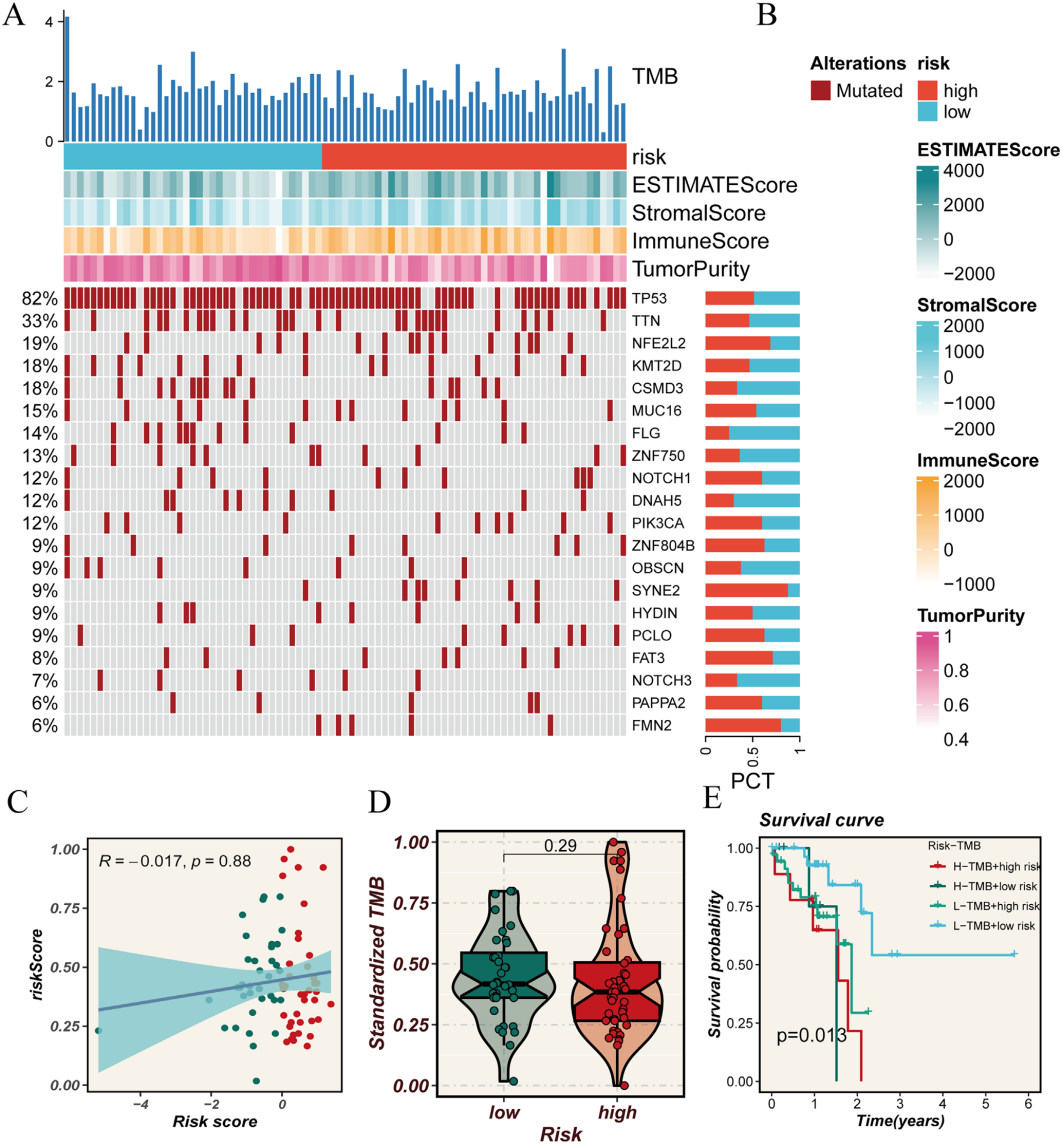
Analysis of tumour mutation burden in different risk score groups. (A, B) Mutation profiles of high‐risk or low‐risk score groups. The mutations of TMB, ESTIMATEScore, StromalScore, ImmuneScore, Tumorpurity and oncogene/tumour suppressor genes were shown by histogram or heat map. The differences are indicated by different colours. (C) Scatter plots showing the correlation between risk scores and standardised TMB. The blue line represents the regression line, and the shaded area represents the confidence interval. (D) Violin charts compare the standardised TMB of the high‐risk or low‐risk score groups. (E) Survival curve analysis after grouping patients based on high/low TMB status and high‐risk or low‐risk scores as factors.

**FIGURE 4:**
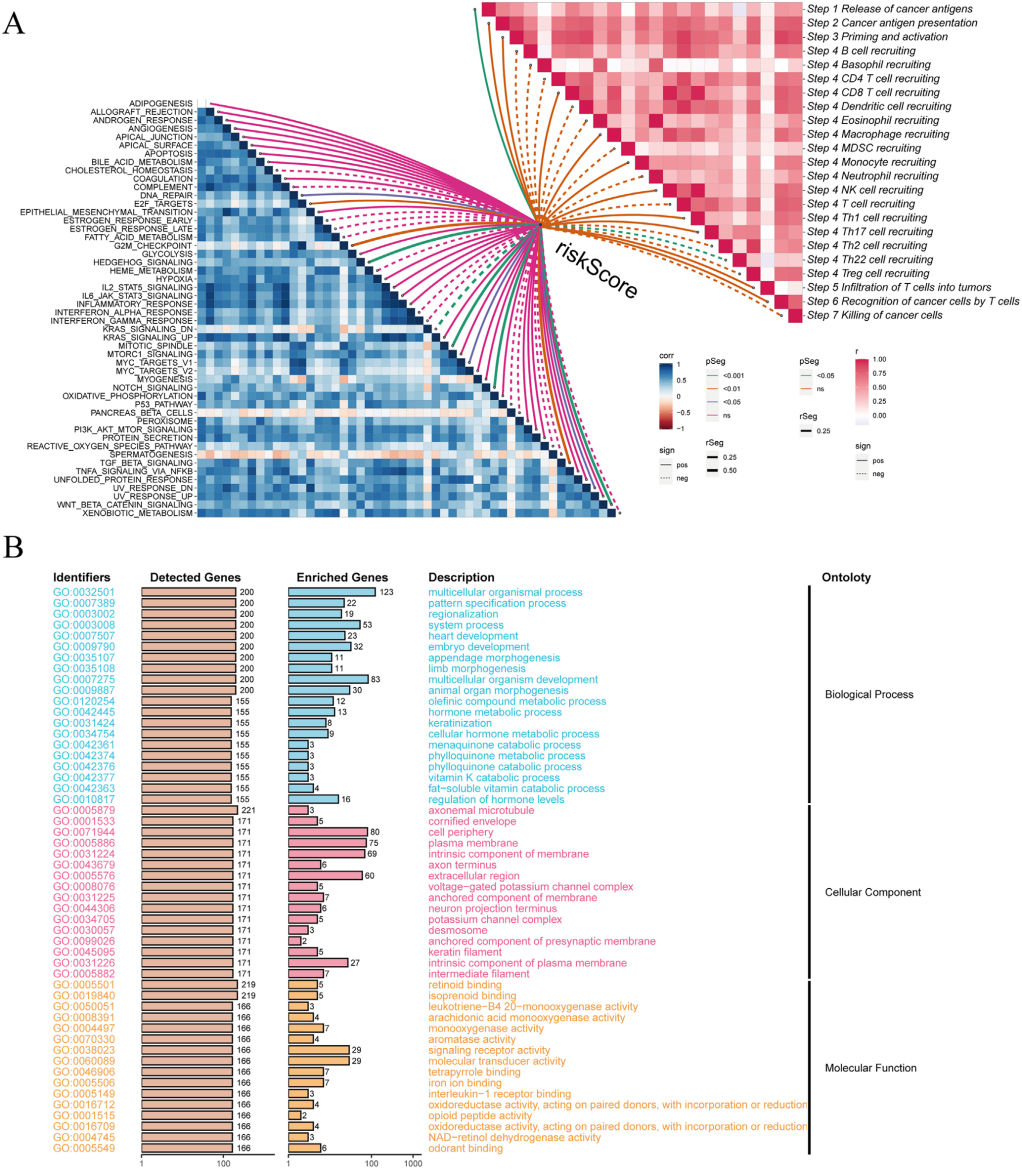
Enrichment analysis of differential genes in high‐risk or low‐risk score groups. (A) Heat maps showing correlation analysis of risk scores with cancer‐related pathways (left) or immune‐related pathways (right). The intensity of blue/red colour in the heat map indicates the correlation coefficient (*r*). The solid connection has a significant positive correlation, and the dotted connection has a significant negative correlation. The different colours of the connecting lines indicate whether they are significant or not, and NS means ‘not significant’. (B) Histogram showing the genetic ontological (GO) enrichment analysis of differentially expressed genes.

The standardised tumour mutation burden (TMB) analysis is shown in Figure 3A, where NFE2L2, NOTCH1, SYNE2, FAT3, PAPPA2 and FMN2 showed lower mutation frequencies in the low‐risk corner. TumorPurity, ImmuneScore, StromalScore and ESTIMATES core are shown in different colours (Figure 3B). There was a positive correlation trend between the risk score and TMB, but unfortunately, this calculation was not statistically significant (Figure 3C). Moreover, after standardising TMB, tumour mutations exhibited a significantly reduced burden in the low‐risk group compared to other groups, which requires further exploration through larger sample statistical analysis. Survival analysis showed that the combination of TMB and risk score had a significant impact on patient survival rate, with lower tumour mutation burden and lower risk score being associated with better survival outcomes. Additionally, we conducted pathway‐related enrichment analysis on glycolipid metabolism genes (Figure 4A,B). Figure 4A presents a heatmap showing the relationship between risk score and immune‐related or tumour‐related pathways. A positive correlation was observed between the risk score and the release of tumour antigens, while a negative correlation was found with Th22 recruitment, and the statistical differences were significant. Additionally, DNA damage repair, HEDGEHOG signalling and MITOTIC‐SPINDLE showed significant positive correlation with the risk score. GO enrichment analysis also indicated that this gene set might affect cancer development by participating in biological processes such as multicellular organismal process or molecular functions such as signalling receptor activity and molecular transducer activity(Figure 4B).

### 3.4 Single‐Cell Sequencing Uncovers the Potential Biological Effects of the Gene Set on ESCC Patients

**FIGURE 5:**
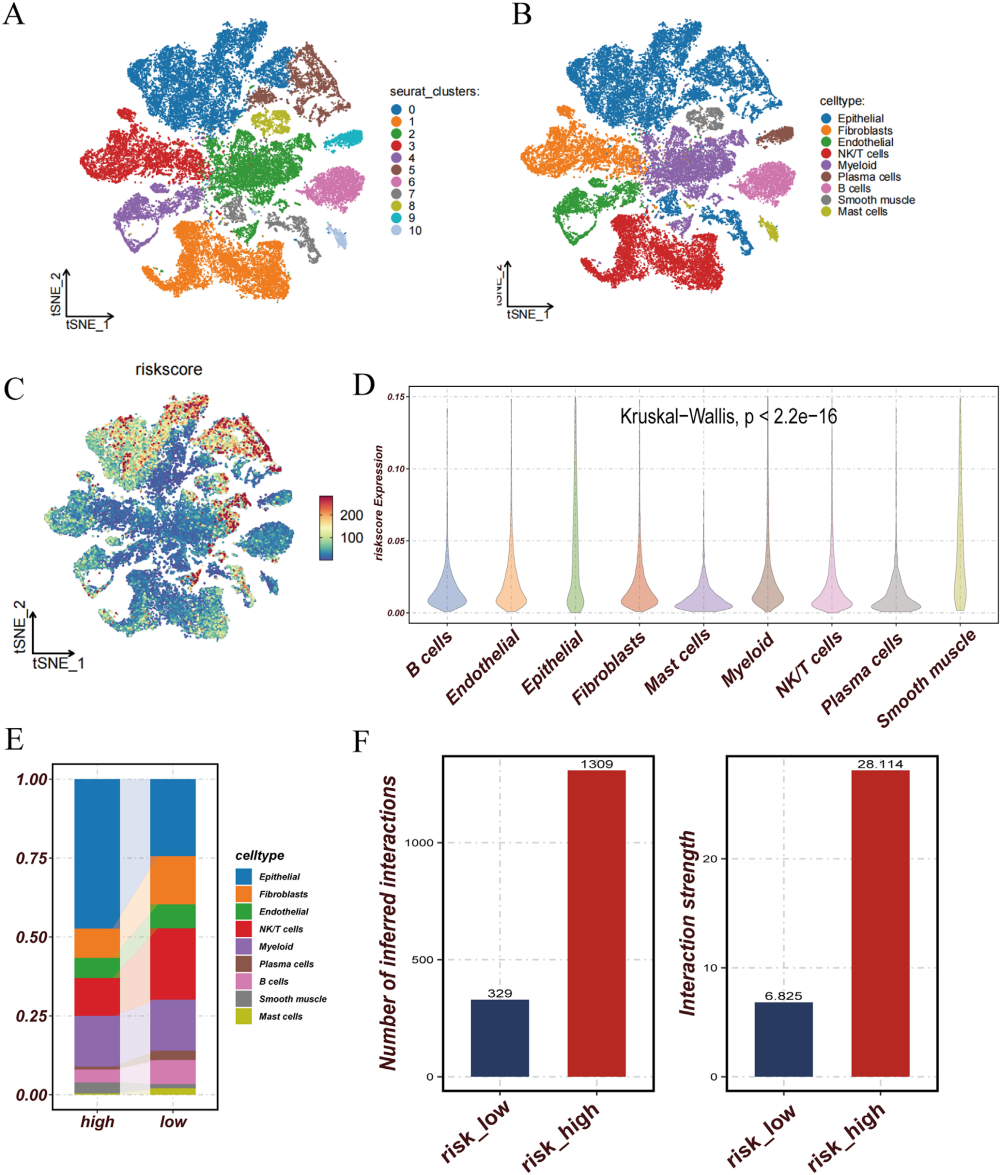
Risk scores associated with various cell types were systematically analysed. (A) t‐SNE map shows clustering of different cell types based on gene expression profiles. (B) t‐SNE map with annotated cell types. (C) t‐SNE plots showing the distribution of risk scores across different cell types. (D) Presentation of risk scores for different cell types. (E) Different proportions of cell types in the high‐risk or low‐risk score groups. (F) Number and intensity of intercellular interactions in the high‐risk or low‐risk score groups.

**FIGURE 6:**
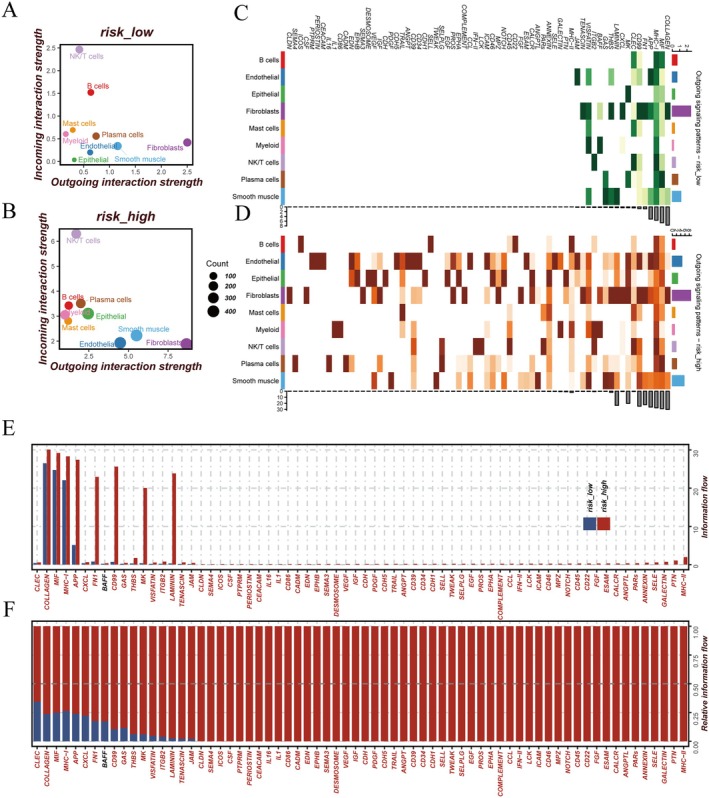
Comparison of cell interactions and communication patterns between high‐risk or low‐risk score groups. (A, B) Scatter plot shows the strength of the incoming interaction and outgoing interaction of each cell type in the low‐risk score (A) and high‐risk score (B) groups. The size of each point reflects the relative amount of action. (C, D) Outgoing signal pattern and strength of each cell type in the low‐risk score group (C) and high‐risk score group (D) with heat maps. (E) Histogram of the absolute information flow of the signal path between the high‐risk score and low‐risk score groups. (F) Histogram of the relative information flow of specific signalling pathways in the high‐risk score and low‐risk score groups. The paths with higher information flow in the high‐risk score group were marked in red, and the paths with higher information flow in the low‐risk score group were marked in blue.

We performed comprehensive single‐cell level analysis using the GSE160269 ESCC dataset. Through dimensionality reduction, clustering and systematic cell annotation, we identified nine distinct cell populations: Epithelial, fibroblasts, endothelial, NK/T cells, myeloid, plasma cells, B cells, smooth muscle and mast cells (Figure 5A,B). Risk score distribution analysis across these cellular populations revealed notable variations, with epithelial and smooth muscle cells demonstrating significantly elevated risk scores (Figure 5C,D). Comparative analysis between risk groups unveiled distinct cellular compositional patterns, characterised by an enrichment of epithelial cells in the high‐risk group and a predominance of NK/T cells in the low‐risk group (Figure 5E). Our intercellular communication analysis revealed that the high‐risk group exhibited more robust and complex cellular interactions (Figure 5F). This enhanced network complexity was evident in both incoming and outgoing communication patterns across cell types (Figure 6A,B). Notably, NK/T cells demonstrated strong incoming interaction signatures, while fibroblasts exhibited pronounced outgoing interaction patterns. The high‐risk group showed particularly distinctive features in signal transmission, with epithelial, endothelial and smooth muscle cells displaying significantly enhanced signalling capabilities compared to their low‐risk counterparts (Figure 6C,D). The relative and absolute information flow analyses of signalling pathways, visualised through bar charts, further emphasised the differential communication intensities across various molecular pathways (Figure 6E,F).

### 3.5 Drug Sensitivity Analysis

**FIGURE 7:**
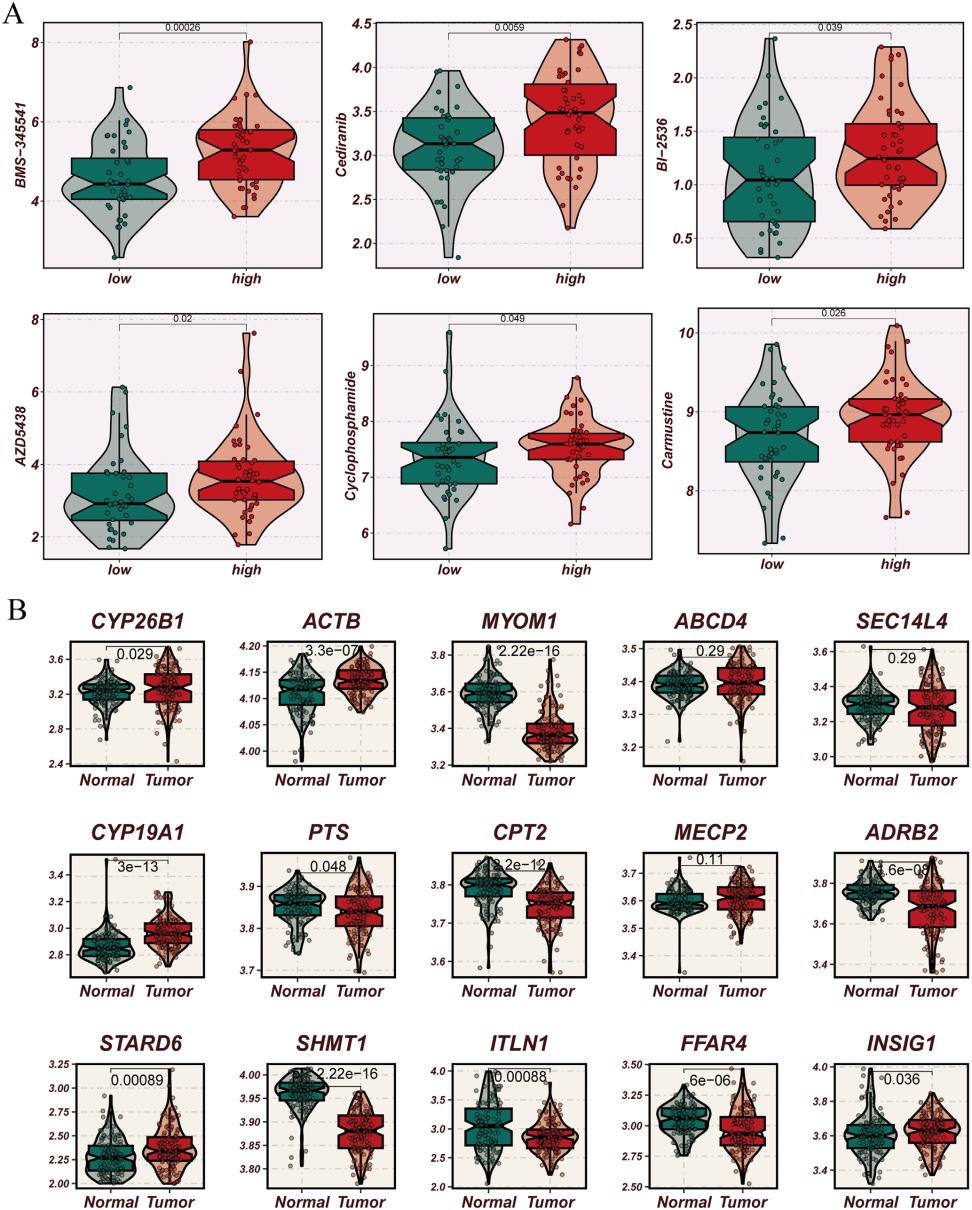
Drug sensitivity analysis of high‐risk or low‐risk score groups and expression difference of 15 core genes in tumour/normal groups. (A) BMS‐345541, Cediranib, BL‐2536, AZD5438, cyclophosphamide and carmustine exhibited different sensitivities in the high‐risk or low‐risk score groups. (B) Expression difference of 15 core genes in the tumour/normal group.

Patients in the high‐risk group demonstrated increased sensitivity to several drugs, including BMS‐345541, Cediranib, BL‐2536, AZD5438, cyclophosphamide and carmustine (Figure 7A). These findings suggest potential therapeutic opportunities for high‐risk patients. The expression profiles of the 15 key genes also showed significant differences between the tumour and normal tissues (Figure 7B).

### 3.6 MECP2 Knockdown Suppresses Cell Proliferation, Colony Formation and Tumour Growth In Vitro and In Vivo

**FIGURE 8:**
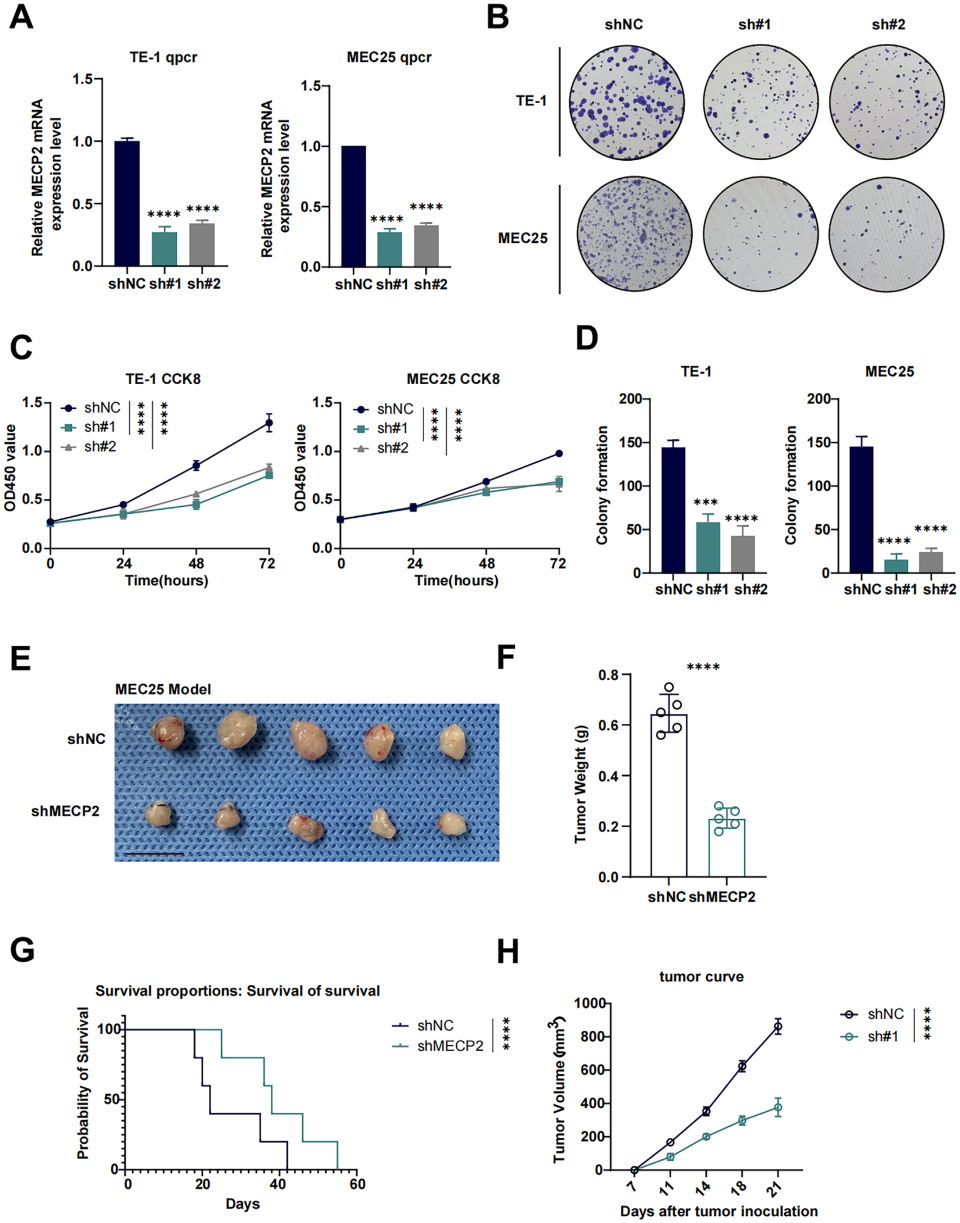
MECP2 knockdown suppresses cell proliferation, colony formation and tumour growth in vitro and in vivo. (A) qPCR analysis of MECP2 mRNA expression: MECP2 expression levels were significantly reduced in TE‐1 and MEC25 cells transfected with shMECP2 constructs (sh#1 and sh#2) compared to the negative control (shNC). Data are presented as mean ± SEM (****p < 0.0001, ***p < 0.001). (B) Colony formation assay: Representative images of colonies formed by TE‐1 and MEC25 cells with MECP2 knockdown (sh#1 and sh#2) or negative control (shNC). (C) CCK‐8 assay: MECP2 knockdown significantly inhibited cell proliferation in both TE‐1 and MEC25 cells over 24, 48 and 72 h compared to shNC. Data are presented as mean ± SEM (***p < 0.001). (D) Quantification of colony formation: MECP2 knockdown (sh#1 and sh#2) led to a significant decrease in the number of colonies formed by TE‐1 and MEC25 cells compared to shNC. Data are presented as mean ± SEM (***p < 0.001, ****p < 0.0001). (E) Representative images of tumours from MEC25 subcutaneous xenograft models: Tumours from the shMECP2 group were visibly smaller than those from the shNC group. (F) Tumour weight measurement: Tumours in the shMECP2 group weighed significantly less than those in the shNC group. Data are presented as mean ± SEM (****p < 0.0001). (G) Survival analysis: Kaplan–Meier survival curves show improved survival rates in mice with shMECP2 knockdown tumours compared to the shNC group. (H) Tumour growth curve: Tumour volume in the shMECP2 group grew significantly slower than in the shNC group over time. Data are presented as mean ± SEM (****p < 0.0001, ***p < 0.001).

To achieve stable knockdown of MECP2, we used lentiviral transduction to construct shMECP2 knockdown cell lines in TE‐1 and MEC25 ESCC cells. RT‐qPCR confirmed successful knockdown, demonstrating a significant decrease in MECP2 mRNA levels in the knockdown groups (sh#1 and sh#2) relative to the negative control (shNC) (Figure 8A). Following the establishment of the knockdown cell lines, a series of phenotypic experiments were conducted to assess the biological effects of MECP2 knockdown. As shown in Figure 8C, MECP2 knockdown significantly inhibited the proliferation of both TE‐1 and MEC25 cells over 24, 48 and 72 h compared to the shNC group. To further assess the impact of MECP2 knockdown on cell growth, a colony formation assay was performed. MECP2 knockdown (sh#1 and sh#2) markedly reduced the colony formation ability of TE‐1 and MEC25 cells compared to the shNC group, as evidenced by both the representative colony images and the quantitative analysis of colony numbers (Figure 8B,D).

In the vivo experiments, we utilised 6‐ to 8‐week‐old female C57BL/6 mice to establish subcutaneous xenograft models with MEC25 cells, aiming to further investigate the impact of MECP2 on the growth of ESCC cells. Compared to the shNC group, the shMECP2 group showed significantly smaller tumour volumes and lower tumour weights (Figure 8E,F, ****p < 0.0001). The shMECP2 group exhibited a significantly prolonged survival time, as shown by the Kaplan–Meier survival analysis (Figure 8G). The tumour growth curves demonstrated a significantly slower rate of tumour volume expansion in the shMECP2 group compared to the shNC group (Figure 8H, ****p < 0.0001).

## 4 Discussion

Glycolysis and lipid metabolism are crucial for tumour cells in terms of energy supply, cell membrane synthesis, antioxidant stress and metastasis [38, 39]. In this study, we utilised multiple bioinformatics analysis methods to identify 15 glycolipid metabolism genes as independent prognostic factors for ESCC patients. Based on the expression of this gene set, a risk scoring rule was established. ESCC patients were divided into high‐risk and low‐risk groups based on the median score. We verified the performance and clinical value of our gene set in predicting patient prognosis. Compared with the low‐risk group, the high‐risk group had a poorer prognosis. A low ImmuneScore typically suggests less accumulation of immune cells in the tumour microenvironment or low immune cell activity. This usually implies a stronger immune escape ability of the tumour, which may be related to the malignancy of the tumour, treatment resistance and thus leads to a poorer prognosis. The reasons for these phenomena may be determined by the interaction between some genes in the gene set and immune cells. For example, the activation of FFAR4 can reduce inflammatory responses by regulating fatty acid metabolism, which may indirectly affect the expression or activity of ICOSLG and TNFRSF4 [40, 41]. FFAR4 regulates T cell activation and immune responses to a certain extent. For example, FFAR4 activation can decrease the production of pro‐inflammatory factors, including TNF‐α and IL‐6, by suppressing NF‐κB activity [42]. CYP19A1 encodes aromatase, which converts androgens into estrogens. Estrogens regulate the immune responses of B cells, T cells and dendritic cells through oestrogen receptors (ERα and ERβ) [43]. ITLN1 (also known as Omentin‐1) is widely believed to have anti‐inflammatory effects and to be involved in regulating the immune response. For example, ITLN1 can reduce macrophage activation by inhibiting the nuclear factor κB (NF‐κB) signalling pathway [44]. ACTB is an important component of the cytoskeleton and is involved in basic cellular processes such as cell morphology, movement, proliferation and division. For example, the migration and aggregation of T cells and macrophages are crucial in immune responses, and ACTB provides necessary structural support in these processes [45]. CYP26B1 is responsible for metabolising vitamin A (retinol), which is essential in the immune system. Vitamin A regulates the functions of immune cells (especially dendritic cells, macrophages and T cells) through its active form (retinoic acid). CYP26B1 affects immune tolerance and immune responses by regulating the level of retinoic acid. Overexpression of CYP26B1 may suppress immune responses, while its deficiency may lead to excessive immune activation [46].

The tumour microenvironment plays a crucial role in tumour progression and treatment response [47, 48]. Through comprehensive analysis, our study revealed distinct characteristics in the high‐risk cohort, characterised by significantly enhanced cellular interactions, intensified signalling networks and increased molecular complexity. This sophisticated intercellular communication network suggests a more intricate regulatory system within the tumour microenvironment. We hypothesise that these elaborate signalling networks predominantly transmit immunosuppressive and regulatory signals, potentially facilitating immune escape mechanisms, promoting treatment resistance and enhancing tumour invasiveness. Notably, our drug sensitivity analysis demonstrated that patients in the high‐risk group exhibited heightened responsiveness to several therapeutic agents, including BMS‐345541, cediranib, BL‐2536, AZD5438, cyclophosphamide and carmustine. This differential drug sensitivity profile in ESCC patients presents an opportunity for more precise therapeutic interventions. Such targeted approaches could potentially maximise treatment efficacy while minimising collateral damage to healthy tissues, ultimately leading to improved clinical outcomes with reduced adverse effects.

MECP2 (Methyl‐CpG‐binding protein 2) is considered one of the most promising therapeutic targets. MECP2 is an important epigenetic regulatory factor that mainly regulates gene expression by binding to methylated CpG dinucleotide regions. MECP2 recruits transcriptional repression complexes (such as HDACs, histone deacetylases) to inhibit the transcription of target genes. MECP2 is also involved in altering the structure of chromatin, making it more compact and thereby affecting gene expression. In normal cells, MECP2 usually plays a role in suppressing the expression of unnecessary genes [49, 50]. MECP2 was first widely known for its role in Rett syndrome, but recent studies have shown that the role of MECP2 in tumour cells may be dual, that is, tumour‐suppressive or tumour‐promoting, depending on the type of tumour, mutation status and the characteristics of the tumour microenvironment. MECP2 may contribute to tumour development in some cancers by modulating the expression of tumour suppressor genes [51]. Abnormal expression or mutation of MECP2 may lead to the silencing of tumour suppressor genes, thereby promoting tumour development. MECP2 may play different or even opposite roles in different tumours. For example, MECP2 has been shown to suppress cell proliferation through the inhibition of the EMT pathway in breast cancer [52]. However, it has also been reported that MeCP2 promotes ubiquitination‐mediated P53 degradation by inhibiting RPL5/RPL11 transcription, thereby promoting breast cancer growth [53]. Abnormal expression of MECP2 is associated with the clinical prognosis of non‐small‐cell lung cancer (NSCLC) patients. MECP2 promotes tumour occurrence by methylating and inhibiting the expression of some key tumour suppressor genes [54]. In colorectal cancer (CRC), the function of MECP2 has also been studied. Research has found that MECP2 may be downregulated in colorectal cancer tissues, and this downregulation is associated with the malignancy of the tumour and poor prognosis [55]. The role of MECP2 in nervous system tumours has also attracted the interest of researchers. Particularly in gliomas (such as astrocytomas), the abnormal expression of MECP2 may be related to tumour proliferation, invasiveness and drug resistance. MECP2 is upregulated in glioma tissues and cell lines. MECP2 promotes glioma progression by activating the Wnt/β‐Catenin signalling pathway [56]. Relatively little research has been done on MECP2 in oesophageal cancer, but given its role in other cancers, MECP2 may also play an important role in the development and progression of oesophageal cancer. According to our findings, MECP2 also contributes to tumour progression in ESCC. Silencing the expression of MECP2 is beneficial for inhibiting the proliferation of ESCC cells, delaying tumour growth and prolonging survival. As an epigenetic regulatory factor, MECP2 can affect the tumour occurrence and development by regulating the methylation status of genes. Utilising epigenetic modification drugs (such as DNA demethylating agents) to regulate the activity of MECP2 may provide new ideas for tumour treatment.

Despite its strengths, this study has several limitations. The relatively small sample size of the TCGA and GEO datasets may limit the generalisability of the findings. To confirm the prognostic utility of the model, validation in larger, independent cohorts is required. Additionally, the specific molecular mechanisms underlying the roles of individual genes within the glycolipid metabolism signature require further investigation. Although MECP2 was confirmed as a therapeutic target in this study, further research is required to investigate its interactions with other metabolic and signalling pathways in ESCC.

Future studies should also focus on integrating multi‐omics data, including proteomics and metabolomics, to gain a more comprehensive understanding of glycolipid metabolism in ESCC. Such approaches could uncover novel therapeutic targets and provide a deeper understanding of tumour heterogeneity. Furthermore, preclinical and clinical studies are needed to evaluate the efficacy of targeting MECP2 and other glycolipid metabolism‐related pathways in ESCC treatment.

## 5 Conclusions

The 15‐gene prognostic model provides a powerful tool for risk stratification and personalised treatment planning, while MECP2 emerges as a promising therapeutic target with broad implications for ESCC management.

## Author Contributions

**Lin Zhu:** methodology (equal), writing – original draft (lead), writing – review and editing (lead). **Feng Liang:** methodology (equal), writing – review and editing (equal). **Xue Han:** methodology (equal), writing – review and editing (equal). **Bin Ye:** conceptualization (equal), data curation (equal), methodology (equal), writing – review and editing (equal). **Lei Xue:** conceptualization (equal), data curation (equal), methodology (equal), writing – original draft (equal), writing – review and editing (equal).

## Conflicts of Interest

The authors declare no conflicts of interest.

## Supporting information


**Appendix S1.** t‐SNE plot illustrating the distribution of genes critical to tumour biological functions across various cell types.

**Appendix S2.** Interactions between different cell types. (A) Interaction network diagram. Different thicknesses of connecting wires represent different quantities and strengths. (B) Heat maps showing the number and intensity of interactions between different cells.

**Appendix S3.** Correlation analysis of 15 selected core genes and risk score.

## Data Availability

This study used public data from an online database. The data can be obtained freely from TCGA (https://www.cancer.gov/ccg/research/genome‐sequencing/tcga) and GEO (https://www.ncbi.nlm.nih.gov/geo/).
