# Supplementary figures and images for: Machine Learning‐Based Glycolipid Metabolism Gene Signature Predicts Prognosis and Immune Landscape in Oesophageal Squamous Cell Carcinoma
Source: J Cell Mol Med. 2025 Mar 22;29(6):e70434. doi: 10.1111/jcmm.70434 (PMC11928743; doi:10.1111/jcmm.70434)

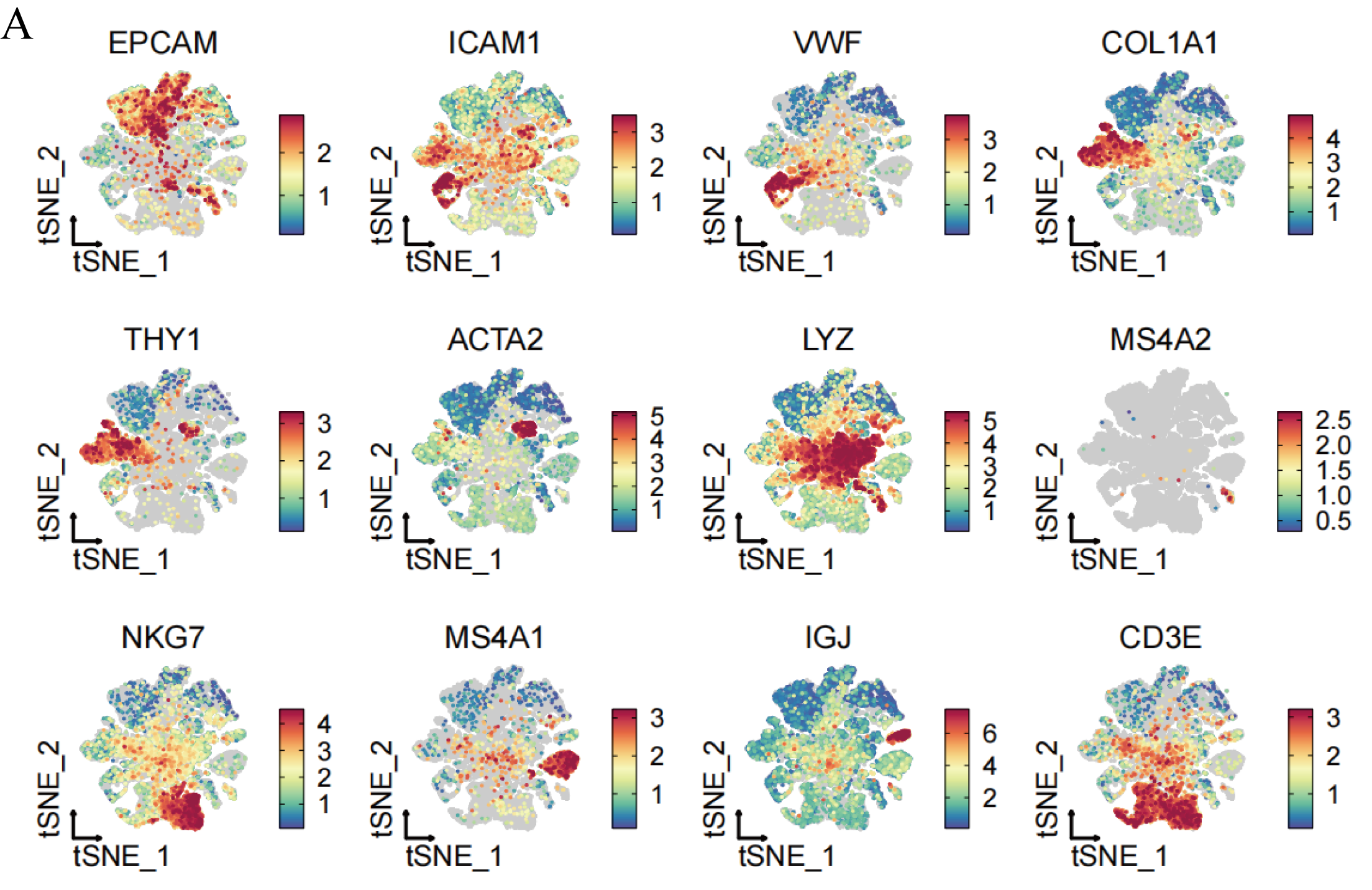

Supplement: Supplementary file 1 — Appendix S1. t‐SNE plot illustrating the distribution of genes critical to tumour biological functions across various cell types. [file JCMM-29-e70434-s002.tif]

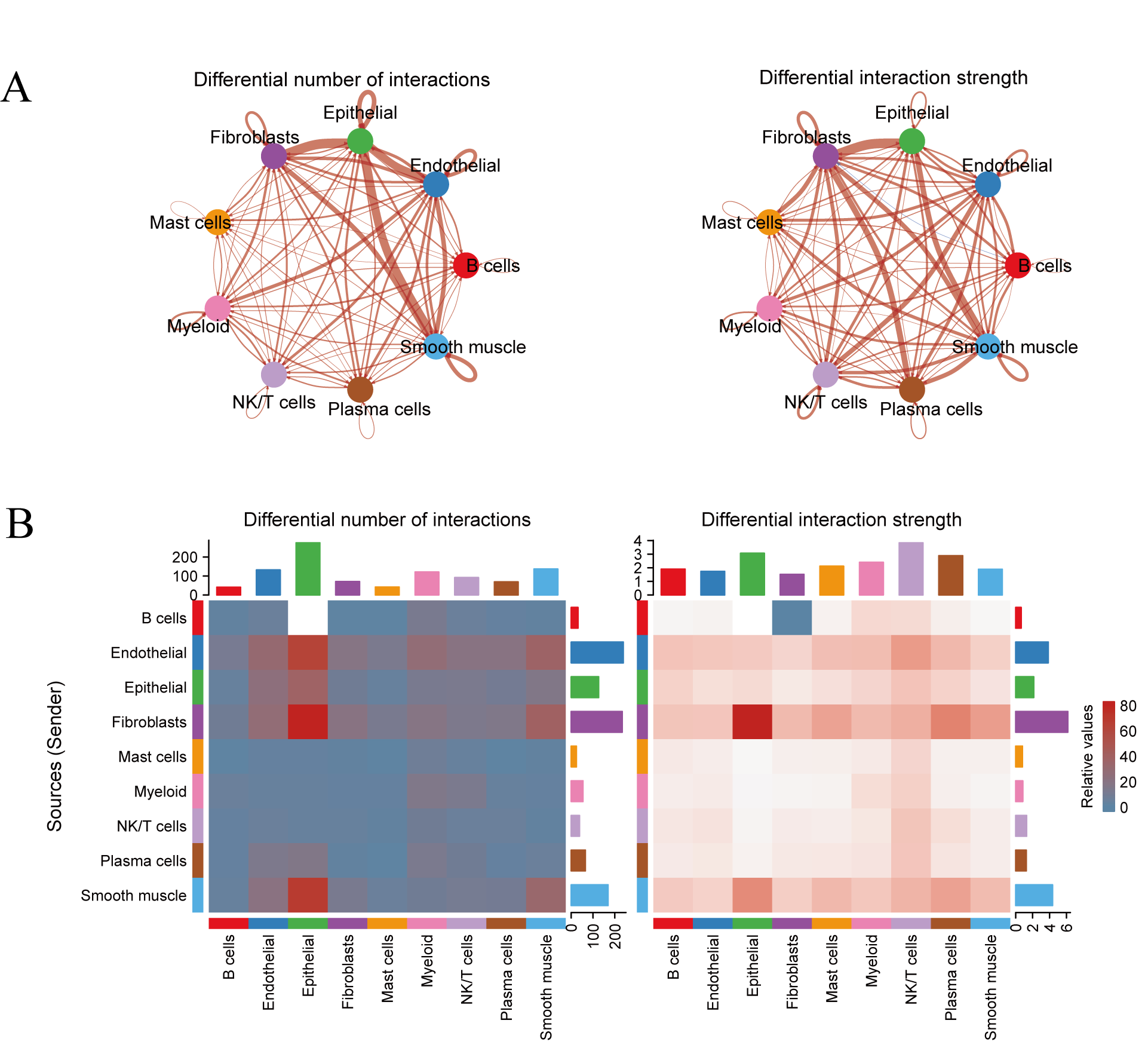

Supplement: Supplementary file 2 — Appendix S2. Interactions between different cell types. (A) Interaction network diagram. Different thicknesses of connecting wires represent different quantities and strengths. (B) Heat maps showing the number and intensity of interactions between different cells. [file JCMM-29-e70434-s001.tif]

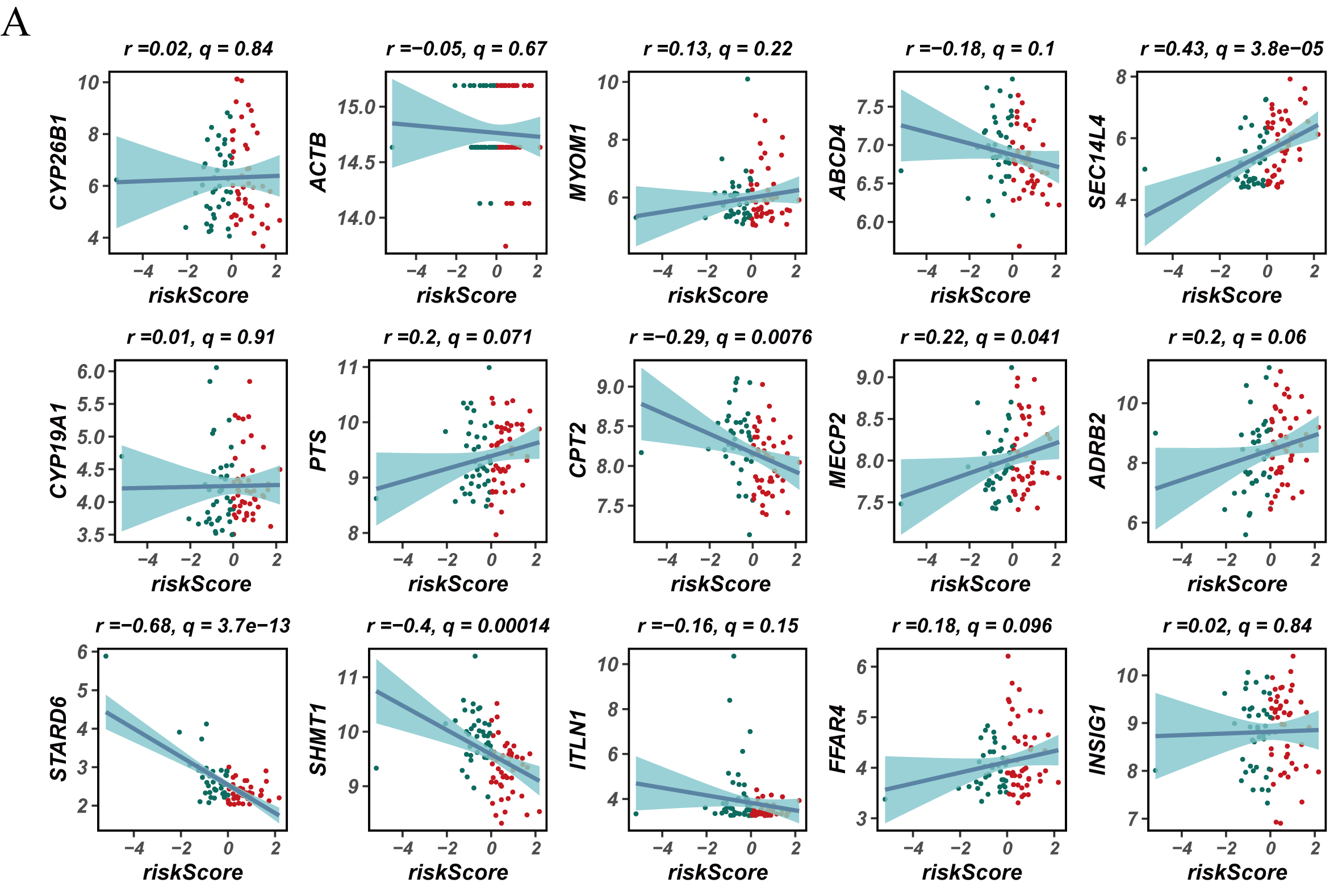

Supplement: Supplementary file 3 — Appendix S3. Correlation analysis of 15 selected core genes and risk score. [file JCMM-29-e70434-s003.tif]
